# It Is Written in the Clot: Coagulation Assessment in Severe Burn Injury

**DOI:** 10.3390/ebj6030037

**Published:** 2025-06-24

**Authors:** Eirini Nikolaidou, Andriana Lazaridou, Christina Iasonidou, Alexandra Tsaroucha, Sophia Papadopoulou, Eleni Kaldoudi, Apostolos Sovatzidis, Despoina Kakagia

**Affiliations:** 1 Department of Plastic, Reconstructive and Hand Surgery & Burns ICU, “George Papanikolaou” General Hospital, 57010 Thessaloniki, Greece; sophipap@hotmail.com; 2 Hematology Department-Hematopoietic Cell Transplantation Unit, Gene and Cell Therapy Center, “George Papanikolaou” General Hospital, 57010 Thessaloniki, Greece; andrianalazaridou@gmail.com; 3 2nd Department of Intensive Care Medicine, “George Papanikolaou” General Hospital, 57010 Thessaloniki, Greece; ciasonidou@gmail.com; 4 Second Department of Surgery, Laboratory of Experimental Surgery, Democritus University of Thrace, 68100 Alexandroupolis, Greece; atsarouc@med.duth.gr; 5 Medical Physics and Medical Informatics, School of Medicine, Democritus University of Thrace, 68100 Alexandroupolis, Greece; kaldoudi@med.duth.gr; 6 Department of Surgery, General Hospital of Giannitsa, 58100 Pella, Greece; t.sovatzidis@gmail.com; 7 Department of Plastic and Reconstructive Surgery, Democritus University Hospital, 68100 Alexandroupolis, Greece; despoinakakagia@yahoo.com

**Keywords:** burns, blood coagulation disorders, blood coagulation tests, blood coagulation factors

## Abstract

Background: Coagulopathy in severe burn injury is associated with complications and mortality. Methods: We compared 3 tests (EXTEM, INTEM, FIBTEM) of rotational thromboelastometry (ROTEM), a viscoelastic coagulation assay (VCA), with conventional coagulation assays (CCAs), fibrinogen, d-dimers and coagulation factors during the five post-burn days in survivors and non-survivors with severe burn injury, in order to correlate the results with burn coagulopathy and prognosis. Results: Seventeen survivors and ten non-survivors, with mean total burn surface area of 33.78% were included. CCAs measurements were abnormal, but unable to detect coagulopathy. At day 2, D-dimers and fibrinogen levels were statistically augmented for non-survivors. Regarding VCAs, FIBTEM MCF increased for non-survivors at day 2 and remained increased for the whole post-burn period. Furthermore, FIBTEM A10 and A20 at day 2 and EXTEM A10, EXTEM A20, EXTEM MCF, and EXTEM CFT at day 5 took abnormal values for the same group (*p* < 0.05). These changes were underlined through abnormal measurements of coagulation factors. Conclusions:CCAs are poor indicators of coagulation status in burn injury, whereas VCAs are more sensitive markers, demonstrating coagulopathy and patients at greater risk of mortality.

## 1. Background

Coagulation disorders are frequently seen in burn patients whose injuries cover a total body surface area (TBSA) >20%, which is defined as severe burn injury [1]. These disorders appear immediately after injury and are divided into two categories: (1) traumatic coagulopathy due to systemic inflammatory response, hypothermia, platelet dysfunction, and tissue injury; and (2) iatrogenic coagulopathy due to resuscitation, hemodilution, and blood loss after surgical excision [2]. Alterations in the coagulation status of severe burn patients are associated with serious complications such as thromboembolic episodes [3], multiple organ failure [4], increased morbidity, and mortality [5,6].

It is imperative to monitor coagulation and the fibrinolytic system in order to understand the importance of early changes in the coagulation status of burn patients. In our armamentarium the tools we have available for the assessment of these disorders are Conventional coagulation assays (CCAs), coagulation factors, Viscoelastic coagulation assays (VCAs). The hemostasis management is usually guided by standard laboratory tests—called CCAs such as such as prothrombin time (pt), activated partial thromboplastin time (ptt), and international normalized ratio (INR) [5]. However, the value of these tests has been questioned due to their prolonged turnaround time and poor bleeding predicting ability [2]. Furthermore, they are indirect indicators of extrinsic and intrinsic clotting pathways without taking into account platelets and fibrinogen levels, which undergo significant changes during the early post-burn period, affecting the equilibrium of the clotting [7,8,9,10]. In addition, the dilution and consumption of coagulation factors are additional aggravating factors in clotting disorders of burn patients [11]. Several studies have shown a decrease in coagulation factors for trauma patients but data in respect of burn patients are sparse [1]. Recently, Viscoelastic coagulation assays (VCAs), such as rotational thromboelastometry (ROTEM), have been increasingly used for timely assessment and management of coagulopathies [12]. These methods are intended to address the weakness of CCAs by analyzing all stages of hemostasis in whole blood samples [12]. By way of a graphical chart, ROTEM provides information about clot formation, clot progress, lysis, and fibrinolysis [13]. In critically ill patients, VCAs are considered the best perioperative monitoring tests, since they can detect both transfusion needs and bleeding disorders [14]. Furthermore, they are successfully used as predictors of mortality in patients with severe trauma [15]. However, despite the fact that these assays are well studied and applied in trauma and critically ill patients, little is known about their application in severe burn patients.

The aim of this study was to compare CCAs (wbc, ht, hb, plt pt, ptt, INR, d-dimer, fibrinogen), coagulation factors (von Willebrand factor, V, VII, IX, X, XI, XII) and VCAs (ROTEM assays, i.e., FIBTEM, EXTEM, INTEM) for survivors and non-survivors during the early post-burn period of five days and to interpret those results as part of the burn coagulopathy.

## 2. Material and Methods

### 2.1. Study Design and Participants

A prospective observational study was conducted at the burn intensive care unit (BICU) of “G. Papanikolaou” General Hospital, Thessaloniki, Greece, from February 2020 to February 2023. The inclusion criteria were adult patients with any type of burn injury with TBSA >20%, admitted at the BICU within the first 24 h from the time of injury. Resuscitation started immediately after the injury according to Parkland Formula. The study was conducted in accordance with the Declaration of Helsinki, and approved by the Institutional Review Board of G. Papanikolaou General Hospital of Thessaloniki, Greece (8/08.01.2020).” Informed consent was obtained from all subjects involved in the study. Patients with other concomitant trauma injuries or coagulation dysfunction, including liver damage, anti-coagulant drugs, antiplatelet agents, or long-term alcoholism, were excluded. Patients who did not survive up to the fifth post-burn day were also excluded. Inhalation injury was defined broncoscopically as an inflammatory reaction in the bronchial mucosa. During the five-day post-burn time period, no patient was treated surgically. Furthermore, the protocol stipulated the administration of blood products only in life threatening situations. No blood products were used, including fresh frozen plasma, red blood cells, albumin, cryoprecipitate.

### 2.2. Blood Sampling

Through radial or femoral arterial catheters with a continuous flush system, arterial blood samples were collected in vacutainer tubes from day 1 to day 5. For the first five post-burn days, patients underwent routine blood and coagulation analyses (pt, INR and ptt, d-dimers, fibrinogen) and viscoelastic coagulation analysis using rotational thromboelastometry (ROTEM Delta; Pentapharm, Munich, Germany). On days 1, 3, and 5, coagulation factors (von Willebrand, V, VII, IX, X, XI, and XII) were analyzed.

### 2.3. CCAs and Cell Counting

Analysis of blood samples was performed using a Sysmex ΧΕ2100 (TOA Medical Electronics, Kobe, Japan) analyzer and included the complete blood cell count (CBC) (red blood cell count (rbc), white blood cell count (wbc), and platelet count (plt)). Conventional coagulation assays (CCAs) (pt, ptt, INR, d-dimers, fibrinogen) were analyzed over the first five post-burn days with an ACL TOP analyzer (IL, Lexington, KY, USA). Normal laboratory reference ranges: ht = 40.00–52.00%; hb = 14.00–18.00 g/dL; wbc = 3800–10,500 count/μL; plt = 150–450 K/μL, INR = 0.8–1.2; pt = 10.00–14.00 s; ptt = 25.00–35.00 s; d-dimers = 0–0.5 μg/mL; fibrinogen = 2.00–4.00 g/L.

### 2.4. Coagulation Factors

Soluble clotting factors (von Willebrand (vWF), FV, FVII, FIX, FX, FXI, FXII) were analyzed on day 1, 3, and 5 (ACL TOP analyzer (IL, Lexington, KY, USA). Immediately after collection, blood samples were transferred to the laboratory and coagulation analysis was started. Factor activity was assessed, given as a percentage of standard activity by comparison of the samples with standard human plasma assays of clotting factors. VWF: Ag was measured using the HemosIL vWF latex enhanced, turbidimetric immunoassay. Normal laboratory reference ranges; von Willebrand = 50.00–200.00 IU/dL, factor V = 50.00–150.00%, factor VII = 50.00–150.00%, factor IX = 60.00–140.00%, factor X = 50.00–150.00%, factor XI = >60%, factor XII = >60%.

### 2.5. ROTEM Parameters and Assays

For ROTEM analysis, the ROTEM Delta System was used. Whole blood samples from day 1 to day 5 were collected and analyzed immediately after collection from the personnel of the 2nd Intensive Care Unit of G. Papanikolaou General Hospital, Thessaloniki, Greece. Each sample was placed in a cuvette. Through a cylindrical pin that is immersed and rotated in the blood, a clot is formed. ROTEM evaluates the clot firmness via the restriction that appears at the moving pin, in an inverse proportional way, by measuring kinetic changes in the clot elasticity [12]. A graphical chart is then extracted with different values: clotting time value (CT, in seconds) depicts the time from the test starting until an amplitude of 2 mm is reached. The clot formation time (CFT, in seconds) is the time taken to achieve a certain level of clot strength, relating to fibrin polymerization and clot stabilization. Finally, a propagation phase though the alpha angle (α, in degrees) assesses the rate of clot formation [13]. Clot amplitude at 10 and 20 min (A10, A20) measures clot firmness at the respective time point after CT. Maximum clot firmness (MCF, in millimeters) measures the maximum displacement and is indicative of the maximum clot strength. Finally, maximum lysis (ML, described as a percentage of MCF) is a measure of the percentage of the decrease in amplitude at the end of the run [13].

Assays of ROTEM that were used: FIBTEM, evaluation of fibrinogen contribution to the blood clot with tissue factor and cytochalasin D, which blocks the contribution of platelets to the maximum clot firmness, leaving the impact of fibrin formation and polymerization; EXTEM, where TF is used to assess extrinsic/common pathways; INTEM, where the coagulation cascade is activated by the intrinsic activator and assesses the intrinsic/common pathway [13,14,15]. Normal ranges according to ROTEM manufacturer: CT = 38–62 s, A10 = 7–23 mm, A20 = 8–24 mm, MCF = 9–25 mm.

### 2.6. Statistical Analysis

Descriptive statistics were calculated using the mean and standard deviation for continuous variables, and frequencies and percentages for categorical variables. Data normality was tested using the Shapiro–Wilk test. Student’s *t*-test was used for comparisons of the means of continuous variables. Levene’s test was used to assess the equality of variances. For non-parametric values, the Mann–Whitney U test was used. For all tests, a *p* value < 0.05 was considered to be statistically significant.Statistical analysis was performed using IBM^®^ SPSS^®^ Statistics Version 29.

## 3. Results

### 3.1. Demographical Data

During the study period, 27 adult patients were enrolled, comprising 15 males and 12 females. The mean cohort age was 58.04 (SD: 16.9) years. A total of 10 deaths occurred (37.04%). Based on survival, two groups were formed (survivors, non-survivors). Ten males and seven females survived, with a mean age of 51.88 (SD: 15.87) years. The mean age for non-survivors was 68.5 (12.99) years and sex was equally distributed. All patients were admitted within the first 24 h and received resuscitation according to the Parkland formula (4 mL × TBSA% × kg).

### 3.2. Type, TBSA, and Depth of Burn Injury

All burn injuries were thermal, apart from two chemical and one electrical burn injury. Total burn surface area (TBSA) ranged from 20% to 71%. Total mean TBSA was 33.78% (SD: 14.56), 32.65% (SD: 10.7) for survivors and 35.7% (SD: 19.28) for non-survivors. Regarding the depth of the burn injury, for survivors, 17.53% (SD: 11.19) of TBSA was second degree and 15% (SD: 12.26) was third degree, whereas for non-survivors 12% (SD: 9.5) was second degree and 23.2% (SD: 18.38) was third degree. The incidence of inhalation injury in our cohort was 25.9%. From the seven patients with inhalation injury, 71.43% died. (Table 1).

### 3.3. Complete Blood Count Results (CBCs)

From the five post-burn days’s CBC results, we compared white blood cell counts (wbc), hematocrit (ht), hemoglobin (hb), and platelets (plt) for survivors and non-survivors. For all of the patients, mean wbc dropped each day from admission to a nadir at day 5. Comparing the two groups, wbc values were higher for non-survivors during the 5-day post-burn period and statistically significant for day 3 (non-survivors; WBC 13,827/μL SD: 5630.89; survivors; WBC 9528.75/μL SD: 4369.88; *p* = 0.04).

Mean hb on admission was 15.27 g/dL (SD: 2.40) for non-survivors and 15.09 g/dL (SD: 2.19) for survivors, and decreased every day. At the fifth post-burn day, mean hb was 9.25 g/dL (SD: 2.28) for non-survivors and 9.74 g/dL (SD: 1.68) for survivors. As expected, ht followed a similar pattern as hb. For days one to three, non-survivors had higher values of ht and hb than survivors, which were statistically significant for day 2 (non-survivors; HT 43.16% SD: 8.63; survivors; HT 37.29% SD: 5.16; *p* = 0.02 and non-survivors; HB 14.15% SD: 2.71; survivors; HT 12.31% SD: 1.74; *p* = 0.03).

Plt showed a downward trend from the first post-burn day to the fifth post-burn day, with the greatest drop in values for non–survivors (non-survivors; day 1 PLT 384.10 K/μL SD: 308.37; day 5 PLT 138.70 K/μL SD: 88.87, survivors; day 1 PLT 236.88 K/μL SD: 80.58; day 5 PLT 184.44 K/μL SD: 71.79, *p* = 0.02, paired sample *t*-test analysis) (Table 2).

### 3.4. Conventional Coagulation Assays (CCAs)

Regarding INR, even though baseline measurements at day 1 were the same between the two groups, for non-survivors they became abnormal from day 2, without being normalized thereafter. This difference between the two groups was statistically significant for days 4 and 5 (non-survivors; INR 1.27 SD: 0.24; survivors; INR 1.05 SD: 0.11; *p* = 0.01 and non-survivors; INR 1.23 SD: 0.28; survivors; INR 1.05 SD: 0.09; *p* = 0.04). The same pattern as for INR emerged for the pt analysis (non-survivors; pt 15.17 s SD: 2.46; survivors; pt 12.48 s SD: 1.25; *p* = 0.04 and non-survivors; pt 14.70 s SD: 3.28; survivors; pt 12.44 s SD: 1.17; *p* = 0.001), whereas for the ptt values, no differences were observed for the two groups. Finally, both d-dimers and fibrinogen levels were augmented for non-survivors for the whole time period, and were statistically different for day 2 (fibrinogen; non-survivors: 4.2 g/L SD: 1.03; survivors: 2.88 g/L SD: 0.94; *p* = 0.03) (Table 3).

### 3.5. Coagulation Factors

Von Willebrand factor was elevated in both groups, being higher in non-survivors than in survivors during the early post-burn period. No critical differences were noted for factor ΙΧ. In contrast, for all other coagulation factors (V, VII, X, XI, XII), non-survivors’ levels were lower than those of survivors, with statistically significant results for day 5 (Table 4).

### 3.6. ROTEM Parameters

FIBTEM MCF at day 2 was increased for non-survivors and was statistically different between the two groups (*p* = 0.04) (Table 5). This parameter remained augmented for non-survivors for the whole post-burn period. Furthermore, for day 2, FIBTEM A10 and A20 were prolonged for the same group (*p* = 0.01 and *p* = 0.01, respectively). Regarding day 5, EXTEM A10, EXTEM A20, EXTEM MCF, and EXTEM CFT were abnormal for non-survivors (*p* = 0.02, *p* = 0.02, *p* = 0.02, *p* = 0.05, respectively). In terms of the clot amplitude at 10 and 20 min, both in FIBTEM and EXTEM it increased in the 5-day period for non-survivors (Table 5 and Table 6). Regarding the INTEM results, apart from CFT1, which took a abnormal value for non-survivors (118.44 s SD: 86.05), all other parameters were within normal values (Table 7).

## 4. Discussion

### 4.1. Demographical Data, Type, TBSA, and Depth of Burn Injury

Non-survivors had a higher mean age (non-survivors: 68.5 SD: 12.99; survivors: 58.04 SD: 16.9) and third-degree TBSA (non-survivors: 23.2 SD: 18.38; survivors: 15 SD: 12.26) compared to survivors. The results of this prospective observational study regarding demographical data, type, TBSA, and depth of burn injury for 27 severe burn patients reinforce the existing knowledge that age and depth of burn areas are predictive factors of mortality. [16]. Total burn surface area (TBSA) ranged from 20% to 71%, but no differences were noticed between the two groups (survivors: 32.65% SD: 10.7 non-survivors: 35.7% SD: 19.28). Regardless of TBSA, patients received the calculated Parkland Formula for the first 24 h. The goal of fluid resuscitation was to achieve and maintain euvolaemia in the face of extensive fluid losses from raw burn areas, profound during the first 24 h following thermal injury. If a burn patient was hypovolemic on arrival, rapidly restoration of intravascular volume was deemed necessary. Once haemodynamic stability was achieved, the maintenance phase of fluid resuscitation begun. Urine output served as an objective indicator of the adequacy of fluid resuscitation. The aim was to maintain a urine output of 0.5–1 mL/kg/h, without the use of diuretics. According to this, every patient was stabilized from the first post-burn day and no burn shock was noticed among our population [17].

Furthermore, the incidence of inhalation injury in our study was 25.9%. Our results are comparable with those reported in other studies, where inhalation incidence rates range between 10 and 20% [18,19,20] and the mortality rate is 25.6% [21]. Through a systematic review of prognostic factors in adults with burn injury, Colohan reported a mortality rate of 27.6% and concluded that the best predictors of mortality among the current published literature on burn prognosis are increased TBSA, increased age, and the presence of inhalation injury [22]. As long as the type of burn injury is concerned, all burn injuries were thermal, apart from two chemical and one electrical burn injury. One of the limitations of our study is that there wasn’t a sub-group analysis of burns other than thermal and their correlation with coagulation disorders. However, even in the literature, most data concern thermal burns. In a recent study of risk factors for the development of thromboembolic events in burn patients, including different types of burns, no differences in outcome was found. Studies focusing on the specific area of interest are undoubtedly required [2].

### 4.2. Complete Blood Count (CBC) Results

Similar trends and several differences were noted among survivors and non-survivors regarding CBC results. Initial systemic inflammation, known to be part of the pathophysiology of burn injuries, leads to increased wbc on admission [23]. Indeed, wbc levels on the first day were the highest among the early post-burn period. Non-survivors’ responses were greater than those of survivors, suggesting that more severe burns in patients trigger greater immune responses to burn-induced inflammation, leading to higher levels of wbc [24]. Over the next few days, wbc levels in both groups decreased. There are two main pathological mechanisms for this decrease: the migration of leukocytes from plasma to burn areas and the suppression of bone marrow following severe burn injury [25,26]. Comparing the two groups, on day 3, non-survivors had statistically significant higher levels of wbc (non-survivors: 13,827/μL SD: 5630; survivors: 9528.75/μL; SD: 4369.88, *p* = 0.04). These results come in accordance several studies that correlate mortality with reduced wbc [27,28]. A limitation of our study regarding wbc fluctuation is that we did not correlate wbc values with infection risk and sepsis, but our study was not designed to answer this question and data regarding infection risk were not collected. Both hb and ht for the two groups were augmented on admission and showed decreasing values in the first five post-burn days. Between groups, there was a statistically significant difference for day 2 for both ht and hb (ht; non-survivors: 43.16%, SD: 8.63; survivors: 37.29% SD: 5.16, *p* = 0.02; hb; non-survivors: 14.15%, SD: 2.71; survivors: 12.31% SD: 1.74, *p* = 0.03). Soman S. et al. examined trends in the components of the CBC in severely burned patients over the first week after injury and concluded that survivors had significantly lower measurements of hb and ht every day from admission to day 7 [28]. The depression of bone marrow production due to inflammation, in combination with the hemolysis of red blood cells, explains the decline [29,30]. Furthermore, the hypovolemic status on admission followed by aggressive fluid resuscitation leads to dilution [27].

Non-survivors’ plt values on admission were higher than those of survivors (plt; non-survivors: 384.10 K/μL, SD: 308.37; survivors: 236.88 K/μL; SD: 80.58). For the first group, they declined and reached their lowest values at day 5, whereas survivors’ lowest values occurred on day 4 and then started to recover. Our findings agree with Osuka a. et al. results who concluded that thrombocytopenia in the early post-burn period is associated with increased mortality [27,28,30,31].

Platelets consumption due to burn injury is mainly responsible for this decline [32].

### 4.3. Conventional Coagulation Assays (CCAs)

Severe burn injury has an impact on the coagulation system. The most common methods of evaluating the coagulopathic status in daily medical routine are the CCAs, such as INR, pt and ptt. They are indirect indicators of extrinsic and intrinsic clotting pathway respectively. The main advantages of CCAs are that they offer a rapid and low-cost assessment of coagulation abnomalties. Laboratory signs of hypocoagulation are associated with mortality in critically ill patients [7]. Likewise; a shortened ptt on admission is associated with an increased risk of in-hospital mortality [2].

Regarding burn injury, the major drawback in the investigation of coagulopathy through CCAs is the absence of a clear definition and defined thresholds. Between studies, the thresholds for INR differ [2]. One study borrowed the term “acute traumatic coagulopathy” from trauma patients in order to describe coagulation disorders in major burn injury with the following thresholds: INR greater than 1.3 and ptt ratio >1.5 times mean normal [33]. Mitra et al. [34] modified the thresholds of INR > 1.5 and ptt > 60 s according to recent studies of acute traumatic coagulopathies [35]. The same rigorous thresholds were used by Kaita et al. [6] and Sherren et al. [36,37], whereas Kang et al. [38], and Muthukumar et al. [35] used cut-off points: INR > 1.2, pt > 14.6 s, ptt > 45 s for acute burn-induced coagulopathy (ABIC). The latest cut-off values are consistent with the international definition of acute traumatic coagulopathy (ATC) given by Davenport et al. [39].

In our study, even though INR baseline was equal for survivors and non-survivors, it increased for non-survivors and remained higher compared to survivors for the whole 5-day period. The same pattern was noted for pt, whereas ptt was within normal ranges for both groups during the first five post-burn days. Even though there was a statistically significant difference in INR between the two groups for days 4 and 5 (INR at day 4; survivors: 1.05 SD: 0.11; non-survivors 1.27 SD: 0.24 *p* = 0.01; INR at day 5; survivors: 1.05 SD: 0.09; non-survivors 1.23 SD: 0.28 *p* = 0.04), values were lower than any of the above definitions of coagulopathy except that of Muthukumar et al.’s study [29]. When the ABIC criteria were applied, both for INR and pt using the cut-off points of 1.2 and 14.6 s, respectively, for days 3, 4, and 5, non-survivors were coagulopathic (for day 3 pt; survivors: 13.10 s SD: 1.64; non-survivors 15.69 SD: 4.54 s; for day 4 pt; survivors: 12.48 s SD: 1.25; non-survivors 15.17 s SD: 2.46 *p* = 0.04; for day 5 pt; survivors: 12.44 s SD: 1.17; non-survivors 14.7 s SD: 3.28 *p* = 0.001). It is clear that different thresholds in the diagnostic criteria of CCAs are responsible for the inconsistency in the results. Furthermore, the changes measured by CCAs and interpreted as coagulopathic, which implies a bleeding tendency. However, looking at the clinical state of the patients and the results of the VCAs, this interpretation must be challenged [10]. Table 8 summarizes the advantages and the disadvantages of each method.

D-dimer and fibrinogen levels reflect fibrinolytic activity. Both d-dimers and fibrinogen concentrations were increased for both groups, but remained elevated for non-survivors. Our results agree with those of several studies in which hypercoagulable status and increased clot formation were found through augmentation of these two parameters [10,40,41]. Martini et al. performed isotope infusion of 1–358 13C-phenylalanine and d5-phenylalanine in severe burn patients to quantify fibrinogen production and consumption, and proved that both fibrinogen synthesis and breakdown were increased after burn injury, but the magnitude of synthesis was larger than that of fibrinogen breakdown, resulting in the increase in fibrinogen availability after burn injury [42]. In our study, fibrinogen levels were significantly increased when compared to the control group throughout the observation period, and were statistically significant at day (non-survivors: 4.2 g/L SD: 1.03; survivors: 2.88 g/L SD: 0.94; *p* = 0.03). This has been described previously and corresponds to increased fibrinogen synthesis and hypercoagulable status [40].

### 4.4. Coagulation Factors

Primary hemostasis initiates through platelet adhesion via von Willebrand factor (vWF) and proceeds to the intrinsic coagulation cascade as it stabilizes coagulation factor VIII [43]. This glycoprotein is also released from the injured endothelium and increases in inflammatory situations [44,45]. In our study, vWF levels were increased in both groups from day 1, with an upward trend for days 3 and 5. Considering that burn injury involves major endothelial damage and activation of the cytokine cascade, enhancing the inflammation response, this augmentation is rational. Furthermore, non-survivors had higher values of vWF for the whole post-burn period. This fact can be associated with a greater TBSA%, deeper burn injury, and worse prognosis. VWF has been associated with inflammatory situations, such as the diagnosis of acute respiratory distress syndrome (ARDS), in which increased levels are associated with bad outcomes, long mechanical ventilation times, and mortality [46]. Although there is no statistical significance in our results, we believe that it is worth mentioned, as Von Willebrand factor was the only Coagulation Factor following this trend. For all other factors, there was a decrease in non-survivors.

Undoubtedly, severe burn injury triggers a greater immune response, leading to burn-induced inflammation [24]. Our study is the first depicting the great impact of severe burn injury in vWF levels. Coagulation factor V levels were lower in admission for non-survivors compared to survivors, whose values were within the normal range and remained decreased for the 5-day post-burn period, and were statistically significant for day 5 (non-survivors: 29.97 SD: 17.10; survivors: 99.22 SD: 33.04; *p* < 0.001). The present study identifies an association between mortality and low levels of factor V. This association is already known for trauma patients, whose increasing traumatic injury severity is associated with decreased factor V antigen levels [47].Regarding burn patients and factor V, our knowledge is limited. Tejiram et al. found normal range factor V activity in all burn patients on admission, but there was no follow-up for further post-burn days [40]. Keyloun et al. evaluated factor V dynamics following burn and nonburn trauma, finding that burn severity was not associated with factor levels, but no correlation for mortality was studied. Since data on factor V in burn patients are limited, indirect conclusions can be drawn from studies of protein C in this population. The endogenous anticoagulant activated protein C (aPC) is a serine protease that inactivates several factors in the coagulation cascade, including factor V. According to a recent study, aPC levels increase from day 3following severe burn injury [48]. Based on these findings, we would expect a decrease in factor V levels, which is consistent with our results. Factor VII was within normal values for both groups but at the lower normal level for non-survivors. Our results are consistent with those of Tejiram et al., who found that admission factor VII remained within normal range and severely burned patients demonstrated decreased mean activity compared with the small burn-size group VII activity (96.2 ± 27.4% versus 70.5 ± 11.9%) [40].

Factors IX, X, and XI for the study period were within normal values for both groups, but non-survivors’ levels were lower compared to those of survivors. Again, these factors have not been studied in relation to burn injury. A recent narrative review evaluating coagulation alterations in major burn patients summarized burn coagulation factors over time and concluded that factor IX is usually within the normal high range during the first 4 post-burn days, with no data for factors X and XI [49]. Finally, factor XII deficiency was observed in both groups and was more severe for non-survivors. Again, little is known regarding this factor in burn patients. For decades, factor XII was considered to have no function for coagulation in vivo, explaining the lack of study interest [50]. Nowadays, we know that even though it does not contribute to “normal” hemostatic fibrin formation at sites of vessel injury, factor XII plays a crucial role in fibrin formation during “pathologic” thrombosis. Low levels of this coagulation factor are associated with thrombosis [51].

### 4.5. ROTEM Parameters

In our study, FIBTEM MCF was increased for non-survivors, indicating an increased fibrinogen concentration. Indeed, at day 2, both FIBTEM MCF and fibrinogen levels were significantly different between the two groups. FIBTEM isolates fibrinogen function using a platelet inhibitor (cytochalasin D), blocking the platelet contribution to clot formation. A FIBTEM MCF in excess of 25 mm suggests an excess of fibrinogen and a procoagulable state [51]. These results are consistent with Schaden et al. results, in which mean FIBTEM MCF was within the reference range 24 h after burn trauma, but increased significantly 48 h afterwards [25]. Furthermore, the same pattern was observed for fibrinogen levels, showing that FIBTEM MCF could be used as a point-of-care assay that is sensitive for fibrinogen function in respect of patients at risk of coagulopathy [51]. Fibrinogen levels were augmented from day 1 to day 5 and, by the end of the 5-day post-burn period, a hypercoagulable status was shown for non-survivors. A recent systematic review on the use of ROTEM values for the diagnosis of coagulopathy, prediction and guidance of blood transfusion, and prediction of mortality in trauma patients, after evaluation of 13 observational studies involving 2835 adult trauma patients, concluded that abnormal FIBTEM clot amplitude and MCF can be used to diagnose acute trauma coagulopathy, predicting the need for massive transfusion, and increased mortality [52]. Furthermore, in a randomized controlled study evaluating ROTEM versus clinical judgment in burn patients undergoing excisional surgery, the use of ROTEM was associated with decreased blood product transfusions [52].

Clot amplitude at 10 and 20 min measures clot strength, with higher values indicating a strengthening clot and a coagulopathy. In our case, non-survivors were more coagulopathic compared to survivors at 5 days post-burn (Table 5 and Table 6). Wiegele et al. performed a 7-day study on burn patients, concluding that there was a 20% increase in clot strength assessed through ROTEM [10]. Comparing ROTEM results with coagulation factors fluctuation, we conclude that the reduced number of coagulation factors is due to increased consumption with the aim of creating stronger clots.

## 5. Conclusions

Several changes occur in patients within the first 5 post-burn days. Our study is the first to assess those changes through CCAs, VCAs, coagulation factors, and CBCs. Even though changes in CCAs exist, they cannot be used to detect burn patients’ coagulopathy. Conversely, VCAs from day 2 to day 5 take abnormal values, especially for non-survivors. These changes are underlined through abnormal values of coagulation factors and fibrinogen products. As a result, CCAs are considered poor indicators of coagulation status in burn injury, whereas VCAs are more sensitive tests in terms of demonstrating coagulation alterations during the early post-burn period and detecting patients who are at greater risk of mortality. Further interventional randomized studies are required to demonstrate the superiority of either approach in the treatment of severe burn injury patients.

## Figures and Tables

**Table 1:** Demographics and burn characteristics (ABSI score = Abbreviated Burn Severity Index score, TBSA = total burn surface area, * = result with statistical significance).

	Total	Survivors	Non-survivors
	27	17	10
Sex	Males: 15 Females: 12	Males: 10 Females: 7	Males: 5 Females: 5
Mean age (years)	58.04 (SD: 16.9)	51.88 (SD: 15.87)	68.5 (12.99) *
Burn type	Thermal: 24 Chemical: 2 Electric: 1	Thermal: 14 Chemical: 2 Electric: 1	Thermal: 10 Chemical: 0 Electric: 0
Mean total TBSA (%)	33.78 (SD: 14.56)	32.65 (SD: 10.7)	35.7 (SD: 19.28)
2nd degree TBSA (%)	15.67(SD: 10.87)	17.53 (SD: 11.19)	12.5 (SD: 9.5)
3rd degree TBSA (%)	18.04 (SD: 15.34)	15 (SD: 12.26)	23.2 (SD: 18.38) *
Inhalation injury	7 (25.9%)	2 (28.57%)	5 (71.43%)

**Table 2:** Complete blood cell counts for survivors and non-survivors for the first five post-burn days (wbc = white blood cell count, ht = hematocrit, hb = hemoglobin, plt = platelets, * = result with statistical significance) (normal laboratory reference ranges: ht = 40.00–52.00%; hb = 14.00–18.00 g/dL; wbc = 3800–10,500 count/μL; plt = 150–450 K/μL). Subscripts correspond to the post-burn day of the samplings.

	Survivors	Non-Survivors	*p*-Value
HT_1(%)_	45.04 (6.15)	46.14 (6.7)	0.30
HT_2(%)_	37.29 (5.16)	43.16 (8.63)	0.02 *
HT_3(%)_	33.52 (4.79)	35.14 (7.16)	0.32
HT_4(%)_	30.20 (7.33)	32.76 (5.81)	0.18
HT_5(%)_	30.11 (5.25)	28.61 (6.80)	0.27
HB_1(g/dL)_	15.09 (2.19)	15.27 (2.40)	0.4
HB_2(g/dL)_	12.31 (1.74)	14.15 (2.71)	0.03 *
HB_3(g/dL)_	10.97 (1.57)	11.62 (2.29)	0.26
HB_4(g/dL)_	11.77 (5.86)	10.77 (1.85)	0.3
HB_5(g/dL)_	9.74 (1.68)	9.25 (2.28)	0.2
WBC_1(/μL)_	18,749.38 (8982.75)	22,467 (6042.72)	0.21
WBC_2(/μL)_	11,747.13 (5562.52)	16,486.33 (8455.71)	0.08
WBC_3(/μL)_	9528.75 (4369.88)	13,827 (5630.89)	0.04 *
WBC_4(/μL)_	8651.25 (3648.58)	13,407 (8381.74)	0.12
WBC_5(/μL)_	8505 (3441.78)	10,211 (6184.88)	0.44
PLT_1(/μL)_	236.88 (80.58)	384.10 (308.37)	0.6
PLT_2(K/μL)_	188.81 (67.56)	293.30 (256.16)	0.22
PLT_3(K/μL)_	161.25 (51.23)	207.90 (186.54)	0.44
PLT_4(K/μL)_	161.68 (52.36)	162 (109.89)	0.99
PLT_5(K/μL)_	184.44 (71.79)	138.70 (88.87)	0.16

**Table 3:** Results of conventional coagulation assays for survivors and non-survivors for the first five post-burn days (pt = prothrombin time, ptt = activated partial thromboplastin time, INR = international normalized ratio, * = result with statistical significance, abnormal values are underlined) (normal laboratory reference ranges: INR = 0.8–1.2; pt = 10.00–14.00 s; ptt = 25.00–35.00 s; d-dimers = 0–0.5 μg/mL; fibrinogen = 2.00–4.00 g/L). Subscripts correspond to the post-burn day of the sampling.

	Survivors	Non-Survivors	*p*-Value
INR_1_	1.12 (0.16)	1.11 (0.28)	0.41
INR_2_	1.16 (0.16)	1.22 (0.33)	0.16
INR_3_	1.10 (0.14)	1.32 (0.42)	0.10
INR_4_	1.05 (0.11)	1.27 (0.24)	0.01 *
INR_5_	1.05 (0.09)	1.23 (0.28)	0.04 *
pt_1(s)_	13.25 (1.83)	13.27 (2.98)	0.46
pt_2(s)_	13.76 (1.95)	14.51 (3.64)	0.91
pt_3(s)_	13.10 (1.64)	15.69 (4.54)	0.07
pt_4(s)_	12.48 (1.25)	15.17 (2.46)	0.04 *
pt_5(s)_	12.44 (1.17)	14.70 (3.28)	0.001 *
ptt_1(s)_	27.69 (4.77)	27.06 (4.38)	0.75
ptt_2(s)_	29.38 (5.65)	33.03 (4.51)	0.19
ptt_3(s)_	33.48 (4.33)	36.07 (7.44)	0.42
ptt_4(s)_	32.19 (4.24)	33.49 (6.27)	0.91
ptt_5(s)_	32.06 (4.02)	36.99 (9.66)	0.28
ddimers_1(μg/mL)_	1.15 (0.89)	1.85 (2.16)	0.35
ddimers_2(μg/mL)_	1.11 (0.99)	1.80 (2.48)	0.42
ddimers_3(μg/mL)_	0.93 (0.89)	0.98 (0.63)	0.88
ddimers_4(μg/mL)_	0.46 (0.17)	0.97 (0.68)	0.82
ddimers_5(μg/mL)_	1.14 (1.02)	1.5 (1.17)	0.42
fibrinogen_1(g/L)_	3.06 (0.98)	3.08 (0.88)	0.96
fibrinogen_2(g/L)_	2.88 (0.94)	4.2 (1.03)	0.03 *
fibrinogen_3(g/L)_	3.74 (0.88)	4.93 (0.94)	0.07
fibrinogen_4(g/L)_	4.74 (1.87)	5.78 (1.68)	0.33
fibrinogen_5(g/L)_	4.65 (1.26)	5.37 (1.69)	0.26

**Table 4:** Coagulation factor measurements for survivors and non-survivors during the first five post-burn days (* = result with statistical significance, abnormal values are underlined) (normal laboratory reference ranges; von Willebrand = 50.00–200.00 IU/dL, factor V = 50.00–150.00%, factor VII = 50.00–150.00%, factor IX = 60.00–140.00%, factor X = 50.00–150.00%, factor XI = >60%, factor XII = >60%). Subscripts correspond to the post-burn day of the sampling.

	Survivors	Non-Survivors	*p*-Value
Von Willebrand_1(IU/dL))_	258.45 (121.91)	295.50 (109.24)	0.46
Von Willebrand_2(IU/dL)_	243.31 (63.1)	333.94 (174.8)	0.2
Von Willebrand_3(IU/dL)_	266.54 (132.7)	323.73 (102.01)	0.35
Factor V_1(%)_	60.12 (25.78)	41.45 (15.89)	0.55
Factor V_3(%)_	80.14 (36.28)	49.12 (20.23)	0.42
Factor V_5(%)_	99.22 (33.04)	29.97 (17.10)	<0.001 *
Factor VII_1(%)_	68.91 (27.13)	56.75 (33.71)	0.34
Factor VII_3(%)_	72.89 (32.92)	61.17 (25.16)	0.4
Factor VII_5(%)_	107.24 (30)	50.78 (30.69)	0.05 *
Factor IX_1(%)_	97.91 (42.67)	80.36 (50.14)	0.36
Factor IX_3(%)_	106.2 (33.48)	112.46 (55.61)	0.79
Factor IX_5(%)_	145.95 (40.85)	119.4 (70.44)	0.33
Factor X_1(%)_	67.90 (21.80)	55.48 (28.06)	0.24
Factor X_3(%)_	73.84 (42.18)	54.35 (28.16)	0.27
Factor X_5(%)_	112.32 (87.27)	45.88 (17.13)	0.05 *
Factor XI_1(%)_	78.62 (27.93)	62.94 (31.17)	0.22
Factor XI_3(%)_	67.89 (21.13)	53.06 (28.11)	0.22
Factor XI_5(%)_	91.02 (19.50)	50.71 (34.33)	0.01 *
Factor XII_1(%)_	53.77 (33.10)	43.87 (21.54)	0.42
Factor XII_3(%)_	36.37 (24.74)	34.82 (16.24)	0.88
Factor XII_5(%)_	49.19 (31.96)	27.94 (9.27)	0.05 *

**Table 5:** FIBTEM assay results for survivors and non-survivors during the first five post-burn days (* = result with statistical significance, abnormal values are underlined) (normal ranges according to ROTEM manufacturer: CT = 38–62 s, A10 = 7–23 mm, A20 = 8–24 mm, MCF = 9–25 mm). Subscripts correspond to the post-burn day of the sampling.

	Survivors	Non-Survivors	*p*-Value
CT_1(s)_	58.93 (15.83)	63.78 (18.03)	0.49
CT_2(s)_	63.88 (18.59)	107.20 (95.82)	0.19
CT_3(s)_	64.059 (15.32)	92.40 (87.39)	0.33
CT_4(s)_	67.33 (14.98)	61.40 (24.19)	0.45
CT_5(s)_	65.50 (9.41)	71.70 (14.86)	0.28
A10_1(mm)_	13.56 (5.41)	15.80 (3.09)	0.2
A10_2(mm)_	13.40 (4.38)	28.13 (4.05)	0.01 *
A10_3(mm)_	22.20 (6.82)	26.65 (10.08)	0.22
A10_4(mm)_	22.00 (6.53)	26.13 (6.08)	0.12
A10_5(mm)_	23.40 (7.59)	27.30 (11.17)	0.37
A20_1(mm)_	16.33 (7.84)	16.79 (4.90)	0.86
A20_2(mm)_	15.20 (4.42)	20.06 (4.37)	0.01 *
A20_3(mm)_	24.30 (7.24)	28.12 (12.38)	0.38
A20_4(mm)_	23.90 (6.70)	28.53 (6.53)	0.10
A20_5(mm)_	25.50 (7.84)	31.50 (8.55)	0.11
MCF_1(mm)_	18.60 (4.17)	18.22 (10.06)	0.89
MCF_2(mm)_	17.70 (4.90)	22.19 (6.24)	0.04 *
MCF_3(mm)_	26.50 (7.98)	33.65 (14.23)	0.15
MCF_4(mm)_	26.60 (7.15)	31.00 (7.02)	0.14
MCF_5(mm)_	27.30 (11.15)	33.80 (9.30)	0.16

**Table 6:** EXTEM assay results for survivors and non-survivors during the first five post-burn days (* = result with statistical significance, abnormal values are underlined) (normal ranges: CT = 38–79 s, CFT = 34–159 s, A10 = 43–65 mm, A20 = 50–71 mm, MCF = 50–72 mm). Subscripts correspond to the post-burn day of the sampling.

	Survivors	Non-Survivors	*p*-Value
CT_1(s)_	65.11 (15.13)	62.06 (19.42)	0.69
CT_2(s)_	75.10 (19.93)	67.94 (33.30)	0.54
CT_3(s)_	62.60 (10.63)	61.35 (10.38)	0.76
CT_4(s)_	73.40 (15.45)	72.53 (20.83)	0.91
CFT_5(s)_	77.00 (25.66)	61.50 (21.51)	0.16
CFT_1(s)_	129.78 (105.92)	106.53 (72.94)	0.53
CFT_2(s)_	102.80 (35.24)	83.58 (22.54)	0.14
CFT_3(s)_	98.38 (33.72)	81.36 (37.83)	0.34
CFT_4(s)_	93.80 (40.05)	74.00 (25.85)	0.14
CFT_5(s)_	96.20 (62.39)	58.10 (12.06)	0.05 *
A10_1(mm)_	48.11 (19.06)	54.67 (10.19)	0.28
A10_2(mm)_	54.10 (8.48)	58.70 (4.87)	0.14
A10_3(mm)_	54.50 (18.85)	57.70 (13.73)	0.61
A10_4(mm)_	54.40 (12.19)	61.33 (7.65)	0.09
A10_5(mm)_	53.80 (13.19)	66.40 (4.94)	0.02 *
A20_1(mm)_	57.67 (12.32)	62.14 (9.11)	0.32
A20_2(mm)_	61.40 (7.31)	65.41 (4.09)	0.13
A20_3(mm)_	64.90 (9.97)	64.06 (13.80)	0.86
A20_4(mm)_	60.90 (12.39)	67.73 (6.34)	0.08
A20_5(mm)_	63.60 (11.27)	72.20 (4.04)	0.02 *
MCF_1(mm)_	60.78 (11.88)	64.27 (7.86)	0.39
MCF_2(mm)_	63.90 (6.23)	67.35 (3.69)	0.13
MCF_3(mm)_	68.20 (7.15)	66.53 (12.60)	0.70
MCF_4(mm)_	61.33 (7.65)	69.60 (5.72)	0.10
MCF_5(mm)_	63.60 (11.02)	73.70 (3.86)	0.02 *

**Table 7:** INTEM assay results for survivors and non-survivors during the first five post-burn days (normal ranges: CT = 100–240 s, CFT = 30–110 s, A10 = 44–66 mm, A20 = 50–71 mm, MCF = 50–72 mm). Subscripts correspond to the post-burn day of the sampling.

	Survivors	Non-Survivors	*p*-Value
CT_1(s)_	187.14 (91.37)	190.22 (89.60)	0.20
CT_2(s)_	179.58 (48.17)	170.80 (34.08)	0.61
CT_3(s)_	178.35 (36.49)	170.10 (46.17)	0.61
CT_4(s)_	197.20 (57.46)	199.70 (38.95)	0.90
CT_5(s)_	210.90 (95.67)	216.70 (54.82)	0.87
CFT_1(s)_	74.92 (28.11)	118.44 (86.05)	0.17
CFT_2(s)_	80.69 (24.30)	90.70 (35.71)	0.40
CFT_3(s)_	72.35 (19.29)	75.30 (23.49)	0.72
CFT_4(s)_	66.93 (20.41)	88.10 (39.74)	0.14
CFT_5(s)_	64.10 (42.55)	87.40 (45.89)	0.25
A10_1(mm)_	51.56 (13.02)	56.17 (6.03)	0.34
A10_2(mm)_	54.80 (8.08)	58.18 (4.45)	0.24
A10_3(mm)_	58.50 (8.17)	59.70 (5.90)	0.65
A10_4(mm)_	55.30 (11.21)	61.86 (7.11)	0.12
A10_5(mm)_	55.40 (12.14)	63.70 (8.98)	0.09
A20_1(mm)_	58.56 (11.32)	63.00 (4.99)	0.29
A20_2(mm)_	61.90 (7.11)	65.18 (4.37)	0.15
A20_3(mm)_	64.10 (7.94)	66.00 (5.60)	0.47
A20_4(mm)_	62.00 (10.62)	67.93 (6.18)	0.37
A20_5(mm)_	61.80 (11.23)	69.30 (7.409)	0.09
MCF_1(mm)_	61.78 (10.64)	65.58 (3.92)	0.33
MCF_2(mm)_	64.40 (5.97)	67.43 (5.02)	0.17
MCF_3(mm)_	66.50 (6.72)	67.17 (5.32)	0.77
MCF_4(mm)_	61.86 (7.11)	69.20 (5.91)	0.17
MCF_5(mm)_	64.30 (10.67)	70.70 (6.83)	0.12

**Table 8:** Comparison of VCAs versus CCAs for Coagulation Assessment of Burn patients. Advantages and disadvantages of each method. (VCAs = viscoelastic coagulation assays, CCAs = conventional coagulation assays, PLTs = platelets).

	CCAs	VCAs
Advantages	- Laboratory assays - Rapid assessment - Commonly used in medical community - Coagulopathic changes for Burn Patients	- Bed-side assays - Holistic assessment of coagulation mechanism (including PLT, coagulation factors, fibrinolysis) - Hypercoagulable status among Burn Patients
Disadvantages	- Indicators of extrinsic and intrinsic clotting pathway, not assessing PLTs and fibrinolysis - Prolonged turnaround time - Absence of a clear definition and defined thresholds for Burn Coagulopathy	- Special instruments required - Need for interpretation of the results

## Data Availability

The data that support the findings of this study are available on request from the corresponding author [E.N.].

## Abbreviations

A10	clot amplitude at 10 min
A20	clot amplitude at 20 min
ABIC	acute burn induced coagulopathy
ABSI score	Abbreviated Burn Severity Index score
aPC	anticoagulant activated protein C
ARDS	acute respiratory distress syndrome
ATC	acute traumatic coagulopathy
AUC	the area under the ROC curve
BICU	burn intensive care unit
CBC	complete blood count
CCAs	conventional coagulation assays
CFT	clot formation time
CT	clotting time value
hb	hemoglobin
HK	high molecular weight kininogen
Ht	hematocrit
INR	international normalized ratio
MCF	maximum clot firmness
ML	maximum lysis
PKK	prekallikrein
Plt	platelets
pt	Prothrombin time
ptt	activated partial thromboplastin time
ROC	receiver operating characteristics
ROTEM	Rotational Thromboelastometry
TBSA	total burn surface area
VCAs	viscoelastic coagulation assays
vWF	VonWillebrand factor
wbc	white blood cells
